# Home-based care practices on prevention of malaria in children under 5 years: A narrative review

**DOI:** 10.1097/MD.0000000000039004

**Published:** 2024-07-19

**Authors:** Emmanuel Ifeanyi Obeagu, Getrude Uzoma Obeagu

**Affiliations:** a Department of Medical Laboratory Science, Kampala International University, Uganda; b School of Nursing Science, Kampala International University, Uganda.

**Keywords:** children, 5 years, home-based care practices, malaria, malaria prevention

## Abstract

Malaria is a major threat to lives in developing countries, especially in Africa. A lot of measures have been tried to curb the increased mortality and morbidity associated with malaria. A lot of resources have been channeled to control the devastating effects of malaria in these parts of the world. The aim of this paper is to discuss home-based care practices on prevention of malaria in children under 5 years. By cutting back on bushes and upholding good hygiene and sanitation, malaria in young children can be prevented in homes. This lessens disease and transmission while also assisting in death prevention and disease reduction. In Africa, Uganda is the third most affected country by malaria, which is a major cause of high morbidity and mortality in young children and pregnant women. This has forced the Government of Uganda and implementing partners, including the Global Fund and the Roll Back Malaria initiative, to redouble efforts to increase the use of insecticide-treated mosquito nets. Effective use of insecticide-treated bed nets is necessary to eliminate the above serious sequelae in children under 5 years old. Households and especially caregivers apply the use of impregnated mosquito nets and cleaning of surrounding bushes. According to research results, the use of indoor residual spray nets and insecticide-impregnated nets has significantly contributed to the prevention of malaria in children.

## 1. Introduction

Malaria remains a global health challenge, particularly in regions where children under 5 years of age bear a disproportionate burden of morbidity and mortality. In the quest for effective strategies to combat malaria, there is a growing recognition of the critical role that home-based care practices play in preventing and mitigating the impact of this devastating disease. This narrative review delves into the nuanced landscape of home-based care practices aimed at preventing malaria in children under 5 years, offering a comprehensive synthesis of existing knowledge and evidence.^[1–7]^ Children in this age group are particularly vulnerable to severe outcomes of malaria due to their developing immune systems. Home-based care, defined as the delivery of healthcare services within the confines of a child’s home environment, presents a promising avenue for malaria prevention. This review aims to explore and analyze a spectrum of home-based interventions, encompassing preventive measures such as bed nets, indoor residual spraying, and antimalarial treatments, with a specific focus on their efficacy and feasibility within the familial and domestic context.^[8–12]^

The cultural, socio-economic, and geographical factors that influence the adoption and success of home-based care practices will be examined to provide a holistic understanding of the challenges and opportunities in implementing these interventions. Additionally, this narrative review will underscore the importance of community engagement, caregiver education, and the role of traditional practices in shaping home-based care strategies.^[13–19]^ As the global health community continues to strive towards the Sustainable Development Goals, with a particular emphasis on ensuring good health and well-being (SDG 3), this review aims to contribute valuable insights to the ongoing discourse on malaria prevention in children under 5. By synthesizing existing evidence, identifying gaps, and proposing recommendations, this narrative review seeks to inform healthcare practitioners, policymakers, and researchers about the potential of home-based care practices in the fight against malaria, ultimately paving the way for more effective and sustainable interventions.

## 2. Methodology

Comprehensive searches were conducted across relevant databases, including PubMed, MEDLINE, Cochrane Library, and other reputable sources. Keywords such as “malaria prevention,” “home-based care,” “children under 5,” and variations were used to identify relevant studies and articles. Inclusion criteria were established to focus on studies published within a specific timeframe, ensuring the inclusion of the most recent and pertinent research.

### 2.1. Use of insecticide-treated mosquito nets and the prevention of malaria in young children under the age of 5

Sociocultural expectations and cultural beliefs that malaria symptoms like fever, backache, nausea, loss of appetite, and vomiting are considered to be signs of pregnancy, prohibiting married women from using insecticide mosquito nets, had an impact on attitudes toward using insecticide-treated mosquito nets as a home-based practice to manage malaria in Tanzania.^[19]^

According to a study by Nuwaha^[20]^ about home-based care practices for malaria prevention in children in the Isingiro district of rural south-western Uganda, households, and especially caregivers, adopt the use of treated nets and clear bushes from the area around the homestead. The study’s findings showed that even though the majority of respondents in rural areas owned insecticide-treated mosquito nets (84%), were aware of the causes of malaria, at least one of its symptoms, and the importance of using insecticide-treated mosquito nets as a preventive measure (over 90%), only 66-point percent were using them regularly.

Hlongwana et al^[21]^ describe a household survey conducted by Knowledge Attitude and Practices with 320 participants in Northern Swaziland. This was the top Knowledge Attitude and Practices in Swaziland, and its purpose was to provide baseline data before a community-level malaria elimination strategy was put into place. Ninety percent of respondents said they use insecticide-treated mosquito nets in their homes to reduce mosquito bites in children under 5, and 99.7% of respondents correctly linked malaria to mosquito bites. Bed net ownership was reported to be 38.8% while indoor residual spraying (IRS) was reported to be 87.2%. The communities that were surveyed had a high level of knowledge about malaria, but neither their preferred information source nor regular community district meetings were providing much information to the populace. According to the study’s findings, the use of indoor residual spraying and insecticide-treated mosquito nets significantly aided in the prevention of malaria in children. Table 1 shows home-based care practices for the prevention of malaria in children under 5.

**Table 1:** Home-based care practices for the prevention of malaria in children under 5.

Practice	Description	Practice	Description
Mosquito nets		Use insecticide-treated bed nets (ITNs) or LLINs during sleep to prevent mosquito bites.	
Indoor residual spraying		Apply insecticides inside the home to create a barrier against mosquitoes.	
Eliminate breeding sites		Regularly remove stagnant water around the home to prevent mosquito breeding.	
Prompt treatment of fever		Seek immediate medical attention if a child has a fever, as it may be a symptom of malaria.	
Antimalarial medication		Administer prescribed antimalarial drugs, especially in high-risk areas or as part of preventive measures.	
Health education		Educate caregivers on malaria symptoms, the importance of prompt treatment, and adherence to medications.	
Nutrition		Provide a balanced diet to enhance the child’s immune system and resistance to infections, including malaria.	
Hygiene practices		Promote regular handwashing and good hygiene to reduce the risk of infections.	
Community engagement		Encourage community participation in malaria prevention efforts for a collective approach.	
Monitoring and surveillance		Regularly monitor and report malaria cases in the community for timely intervention and prevention strategies.	

ITNs = insecticide-treated bed nets, LLINs = long-lasting insecticidal nets.

### 2.2. The effect of wearing protective clothing on preventing malaria in children under 5

O’Meara et al’s study^[22]^ stated that for a significant drop in malaria, home-based care practices and socio-economic conditions needed to be improved to eliminate malaria in Europe and North America. The study recommended that the young should wear protective clothing and that local communities should be made aware of the need to combat poverty because it is closely related to malaria by organizing the community into cooperatives that generate income, thereby enhancing the members’ quality of life. Additionally, educating students about malaria in the classroom can result in the message being implemented at home and shared with other family members, which could have a significant impact in Ruhuha settings. This is similar to what was discovered in Thailand,^[23]^ where a study showed how school kids served as messengers for a variety of community-based anti-malaria actions, such as using full protective clothing for children during the evening and clearing the home environment by clearing bushes around the homesteads to prevent mosquito lodges.

### 2.3. The effect of clearing bushes around homesteads on malaria prevention in children under 5 years

A study by Nyachongi^[24]^ looked at the effectiveness of clearing the brush as a malaria prevention strategy for kids on Rusinga Island in western Kenya. The study found that, of the 1451 people interviewed, 43% were aware of and used bush clearing as a malaria prevention strategy, respectively. Sixty nine percent of people acquired this knowledge through formal education. There was a great deal more gambiae mosquitoes in aquatic habitats exposed to sunlight as opposed to those hidden by vegetation

Karema et al claim that^[25]^ examined the successful malaria prevention strategies used by the local Rwandan communities. Malaria is preventable, according to their study. Families use a variety of preventative measures to keep their homes clean, clear bushes, close their windows early in the evening, avoid spending too much time outside in the late evening, and always sleep under a mosquito net at night. The study came to the additional conclusion that keeping a positive home environment helped Rwandan children under the age of 5 to successfully combat malaria.

By cutting back on bushes and upholding good hygiene and sanitation, malaria in young children can be prevented in homes. This lessens disease and transmission while also assisting in death prevention and disease reduction.^[26]^ Microscopy is used to make a conclusive diagnosis of malaria, including the parasite count and species of the disease. Rapid diagnostic tests have become a more affordable and simpler alternative because they can be used in health centers or communities with little to no equipment or training.^[27]^ Artemisinin, an approach based on artemisinin, is the most effective malaria treatment currently available. Antimalarial drug resistance is a persistent issue. Poor compliance or inappropriate antimalarial use are just two of the many factors that contribute to this issue. Drug resistance has led to an increase in the use of combination therapy, which can also shorten the course of treatment.^[28–30]^

## 3. Recommendations

Policymakers and public health authorities should consider integrating effective home-based care practices into existing malaria control programs. This integration can enhance the reach and impact of interventions targeting children under 5, leveraging the familiarity of home environments. Initiatives should be developed to educate caregivers and communities about the importance of home-based care practices for malaria prevention. Community engagement programs can empower caregivers with the knowledge and skills needed to implement preventive measures effectively. Interventions should be context-specific, considering cultural, socio-economic, and geographical factors that influence the adoption of home-based care practices. Tailoring strategies to local contexts increases the likelihood of acceptance and sustainability. Develop and implement innovative communication strategies, including multimedia tools and community-based campaigns, to disseminate information about home-based care practices. Utilize culturally sensitive and locally relevant approaches to enhance message effectiveness.

Foster collaboration between formal healthcare systems and traditional healers. Traditional practices may influence health-seeking behaviors; therefore, integrating traditional healers into malaria prevention efforts can enhance community acceptance. Conduct longitudinal studies to assess the long-term sustainability and effectiveness of home-based care practices for malaria prevention. Longitudinal data will provide insights into the durability of interventions and their impact on reducing malaria incidence in the targeted age group. Encourage multisectoral collaboration involving health, education, and community development sectors to address the multifaceted nature of malaria prevention. Coordinated efforts can lead to comprehensive interventions that go beyond healthcare facilities.

Establish robust monitoring and evaluation frameworks to assess the performance of home-based care programs. Regular assessments should include indicators related to intervention coverage, compliance, and health outcomes, enabling data-driven decision-making. Prioritize research on the implementation strategies of home-based care practices. Understanding the facilitators and barriers to effective implementation will inform the development of scalable and sustainable interventions. Advocate for policy support at national and international levels to prioritize and integrate home-based care practices for malaria prevention in children under 5. Policymakers should be informed about the potential impact of these practices on reducing childhood malaria.

## 4. Conclusion

The narrative review on “Home-based care practices on prevention of malaria in children under 5 years” provides valuable insights into the current state of knowledge and evidence regarding interventions aimed at preventing malaria in this vulnerable age group within the home environment. The synthesis of existing literature reveals a diverse array of home-based care practices, ranging from bed nets and indoor residual spraying to caregiver education and community engagement initiatives. The review underscores the significance of home-based care as a crucial component in the fight against childhood malaria, particularly in regions where children under 5 bear a disproportionate burden of the disease. The integration of effective preventive measures into the familiar setting of home environments has the potential to enhance coverage and compliance, addressing barriers associated with healthcare facility access and utilization.

Cultural, socio-economic, and geographical factors have been highlighted as critical determinants influencing the success of home-based care practices. Recognizing the importance of tailoring interventions to local contexts, incorporating traditional healing practices, and engaging communities in the decision-making process emerge as key considerations for the development and implementation of effective strategies. In essence, this narrative review serves as a call to action, a call to prioritize home-based care practices within the broader framework of malaria prevention efforts. By doing so, we can work towards creating a future where children under 5 are not only better shielded from the devastating impact of malaria but also empowered to thrive in healthy, supportive home environments.

## Author contributions

**Conceptualization:** Emmanuel Ifeanyi Obeagu.

**Methodology:** Getrude Uzoma Obeagu.

**Resources:** Emmanuel Ifeanyi Obeagu.

**Supervision:** Emmanuel Ifeanyi Obeagu.

**Visualization:** Emmanuel Ifeanyi Obeagu.

**Writing** – **original draft:** Emmanuel Ifeanyi Obeagu, Getrude Uzoma Obeagu.

**Writing** – **review & editing:** Emmanuel Ifeanyi Obeagu, Getrude Uzoma Obeagu.
